# Fungal burden, dimorphic transition and candidalysin: Role in *Candida albicans*-induced vaginal cell damage and mitochondrial activation *in vitro*

**DOI:** 10.1371/journal.pone.0303449

**Published:** 2024-05-20

**Authors:** Luca Spaggiari, Andrea Ardizzoni, Francesco Ricchi, Natalia Pedretti, Caterina Alejandra Squartini Ramos, Gianfranco Bruno Squartini Ramos, Samyr Kenno, Francesco De Seta, Eva Pericolini

**Affiliations:** 1 Clinical and Experimental Medicine PhD Program, University of Modena and Reggio Emilia, Modena, Italy; 2 Department of Surgical, Medical, Dental and Morphological Sciences with Interest in Transplant, Oncological and Regenerative Medicine, University of Modena and Reggio Emilia, Modena, Italy; 3 Institute for Systemic Inflammation Research, University of Lübeck, Lübeck, Germany; 4 Department of Medical Sciences, University of Trieste, Trieste, Italy; 5 Institute for Maternal and Child Health-IRCCS, Burlo Garofolo, Trieste, Italy; University of Jeddah, SAUDI ARABIA

## Abstract

*Candida albicans* (*C*. *albicans*) can behave as a commensal yeast colonizing the vaginal mucosa, and in this condition is tolerated by the epithelium. When the epithelial tolerance breaks down, due to *C*. *albicans* overgrowth and hyphae formation, the generated inflammatory response and cell damage lead to vulvovaginal candidiasis (VVC) symptoms. Here, we focused on the induction of mitochondrial reactive oxygen species (mtROS) in vaginal epithelial cells after *C*. *albicans* infection and the involvement of fungal burden, morphogenesis and candidalysin (CL) production in such induction. Bioluminescent (BLI) *C*. *albicans*, *C*. *albicans* PCA-2 and *C*. *albicans* 529L strains were employed in an *in vitro* infection model including reconstituted vaginal epithelium cells (RVE), produced starting from A-431 cell line. The production of mtROS was kinetically measured by using MitoSOX^™^ Red probe. The potency of *C*. *albicans* to induced cell damage to RVE and *C*. *albicans* proliferation have also been evaluated. *C*. *albicans* induces a rapid mtROS release from vaginal epithelial cells, in parallel with an increase of the fungal load and hyphal formation. Under the same experimental conditions, the 529L *C*. *albicans* strain, known to be defective in CL production, induced a minor mtROS release showing the key role of CL in causing epithelial mithocondrial activation. *C*. *albicans* PCA-2, unable to form hyphae, induced comparable but slower mtROS production as compared to BLI *C*. *albicans* yeasts. By reducing mtROS through a ROS scavenger, an increased fungal burden was observed during RVE infection but not in fungal cultures grown on abiotic surface. Collectively, we conclude that CL, more than fungal load and hyphae formation, seems to play a key role in the rapid activation of mtROS by epithelial cells and in the induction of cell-damage and that mtROS are key elements in the vaginal epithelial cells response to *C*. *albicans*.

## Introduction

*Candida albicans* (*C*. *albicans*) is one of the most known human fungal pathogens. It is responsible of significant clinical conditions, spanning from mild mucosal to severe invasive infections. This species has been recently included by WHO within the critical priority group of pathogenic fungi [1].

*C*. *albicans* is a part of the vaginal microbiota of healthy women: when occurring in low numbers it is normally tolerated as a commensal yeast on the mucosal surface, where it does not trigger any epithelial immune response [2, 3]. However, in immunocompromised hosts or in specific clinical conditions such as VVC that can occur in immunocomopetent women, *C*. *albicans* behaves as an opportunistic pathogen increasing the local fungal burden and its virulence; these events in turn may exceed the tolerance threshold of epithelial cells thus causing an intense inflammatory response, which is the main responsible of the vulvovaginal candidiasis (VVC) symptoms [2]. Such tolerance threshold is defined as tolerability levels of human cells host to Candida presence without triggering an inflammatory response [2]; it varies among women according to several individual factors (i.e. presence of lactobacilli, pH, estrogen levels and many others).

Among mucosal infections, VVC is a very common condition in healthy women in their reproductive age. Therefore, here *C*. *albicans* behaves like a primary pathogen.

*In vitro* studies shown the involvement of several components during the vaginal epithelial cells response to *C*. *albicans* and it is more and more evident that epithelial cells, in addition to their role as mechanical barriers, are capable to polarize host response against infections [4].

Host mitochondria play a crucial role in the innate immune responses by several mechanisms, such as Reactive Oxygen Species (ROS) production [5]. The latter include many derivatives of molecular oxygen, such as hydrogen peroxide H_2_O_2_ (prototype of the group of “two-electron non-radical ROS”) and the superoxide anion radical O_2_*^-^ (as prototype of the group of “free radical ROS”). The major endogenous enzymatic sources of O_2_*^-^ and H_2_O_2_ are transmembrane NADPH oxidases (NOXs) [6–8] and the mitochondrial electron transport chain (ETC) [9], as well as various other sources. At physiological conditions, mitochondrial ROS (mtROS) production promotes the so-called “oxidative eustress”, responsible of cell differentiation, proliferation, migration and vasodilation [10–12]: under these conditions, cells regulate mtROS levels by maintaining a balance between their production and their elimination, avoiding thus their harmful effects [13, 14]. Indeed, mitochondrial activation is also one of the primary responses of the host cells when they are subjected to different external stimuli (including microbes), and such activation is important for the regulation of innate and adaptive immune response [15]. Activation of TLRs 1, 2, and 4 leads to the recruitment of mitochondria to macrophage phagosomes, where they enhance mtROS production [16]. It has been reported that methicillin-resistant *Staphylococcus aureus* (MRSA) induces the generation of mitochondria-derived vesicles containing mtROS that, in turn, help the clearance of intracellular bacteria via TLR signaling [17]. Furthermore, oral epithelial cells treated with candidalysin (CL) showed a rapid production of mtROS, triggering numerous cellular stress responses that ultimately lead to oral epithelial cell necrotic death [18]. The activation of mtROS in response to different microbial infections has been demonstrated to modulate host cells proliferation, vitality and death and to improve the antimicrobial function of the innate immune cells [19]. Interestingly, type I interferon pathway seems to play a key protective role, depending on the time and species, in epithelial response against *Candida* infection through mitochondrial activation [4, 20, 21].

However, excessive amounts of mtROS can cause “oxidative distress”, that results in degradation of intracellular lipids, proteins and DNA, which in turn lead to cell damage and triggers several cell death patterns [22–24]. It has been recently reported that *C*. *albicans* causes cellular oxidative stress and cell death upon activation and mtROS production [25].

During VVC immunopathogenesis, human cells, fungi, and microbiota alteration, all contribute to the disease onset. Therefore, *C*. *albicans* virulence in the vaginal environment is also mediated by its capacity to proliferate, form hyphae, and produce toxic molecules such as Secreted Aspartyl Proteinases (SAPs) and CL (the latter produced only by hyphal forms), that seem to play a key role in the immunopathogenesis of VVC [4, 26, 27]. In particular, CL has been recently shown to induce a potent epithelial cell damage and inflammation both *in vitro* and in a murine model of VVC [28, 29]. Indeed, challenge *C*. *albicans* lacking hyphal-associated gene ECE1 or CL deletion mutant strains in a murine model of VVC resulted in a reduction of the immunopathology, including a decreased proinflammatory cytokines production, neutrophils recruitment and tissue damage, as compared to the challenge with the WT strain. Given that, the CL mutants still robustly form hyphae in vaginal lumen, these results clearly show that hyphal growth is required but not sufficient for VVC immunopathology development. CL is likely the virulence factor that drives these responses [30].

Here we focus on the role of three main factors such as fungal load, morphogenesis and CL in the induction of the epithelial response to *C*. *albicans* by RVE infection model *in vitro*. The production of mtROS and cell damage will be analysed since we observed, in our preliminary data, that *C*. *albicans* induces a time-dependent production of mtROS in vaginal epithelial cells. Interestingly, the morphology, CL, and fungal load are all regulators of mtROS production and cellular damage.

## Results

### Mitochondrial activation by BLI-Ca

We started by analyzing the mtROS production in response to Ca infection, using the RVE infection model [31, 32], both by BLI-Ca yeasts and preformed hyphae with low Multiplicity Of Infection (MOI) 1:1 and high MOI 1:5 (epithelial cells: *Candida*) [33]. As far as we know, this is the first time that mtROS production by RVE is monitored kinetically after Ca yeasts or hyphae infection. Therefore, we chose 5 min reading cycles up to 12 h to collect as much data as possible to describe the phenomenon.

**Fig 1:**
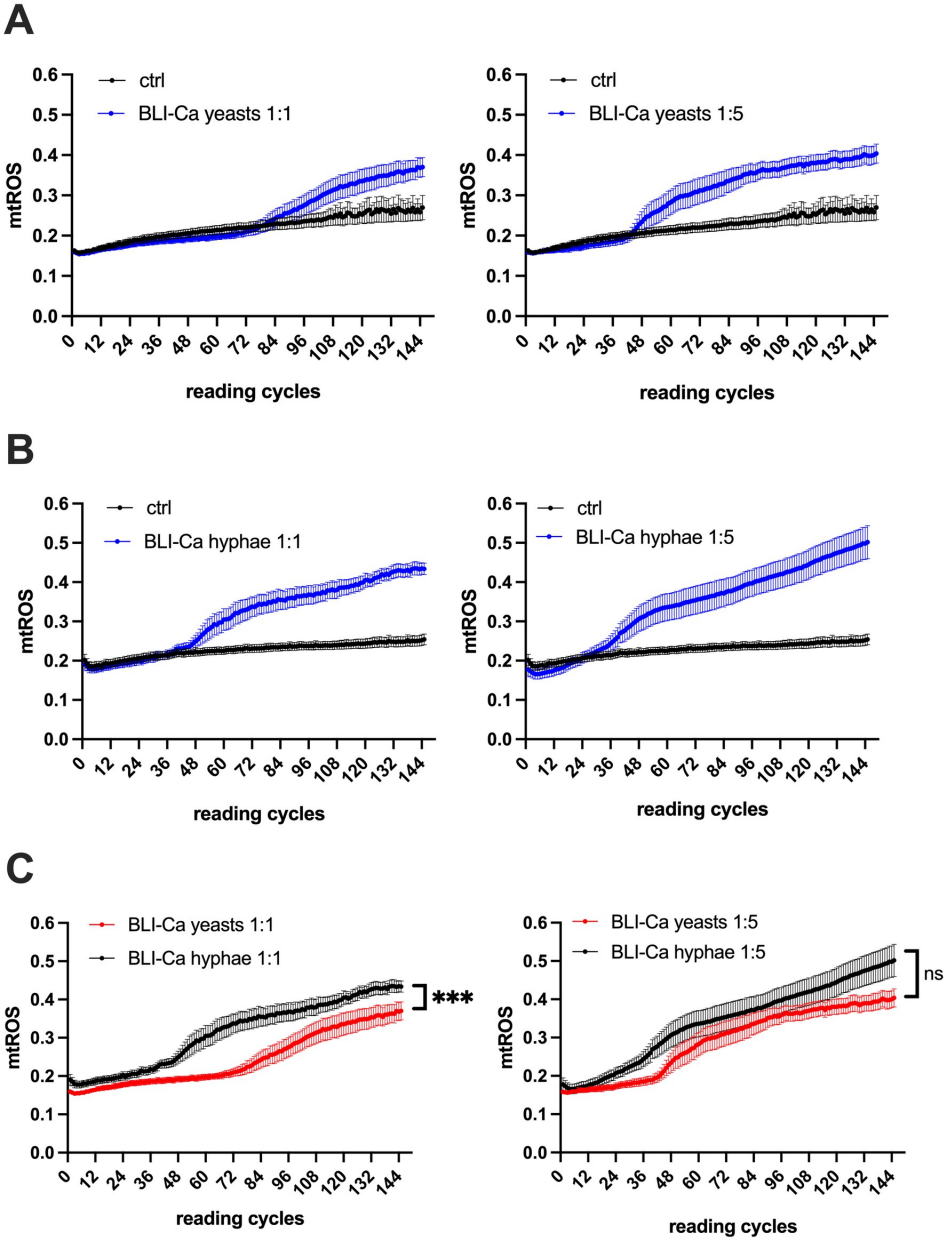
Kinetics of mtROS production by BLI-Ca infected RVE. RVE has been infected with BLI-Ca in yeasts (**A**) or hyphae (**B**) either at MOI 1:1 (left panels) or MOI 1:5 (right panels). In (**C**) the comparisons between mtROS production by BLI-Ca yeasts and hyphae at MOI 1:1 (left panel) and MOI 1:5 (right panel) is shown. The mtROS production was evaluated by fluorescence emission after addition of MitoSOX^™^ Red probe, as detailed in Materials and Methods section. Reading cycles were perfomed at 5 minutes intervals. Ctrl = uninfected RVE. Data are expressed as mean ± SEM. Data are from at least 3 different experiments performed in triplicate. Statistical analysis was performed by using Mann-Whitney U test ***p < 0.001.

Our results show that at MOI 1:1 (epithelial cells/Candida) the mtROS occurred at around 7 h post-infection (84 reading cycles) with yeasts and at around 3.5 h post-infection (40 reading cycles) with hyphae (Fig 1A and 1B—left panels and Fig 4A—left panel). Differently, the response to infection at MOI 1:5 was quicker, i.e., at 3.5 h for yeasts (42 reading cycles) and at 2.5 h for hyphae (30 reading cycles) (Fig 1A and 1B—right panels and Fig 4A—right panel).

A side-by-side analysis of epithelial response to Ca yeasts and hyphae shows that at MOI 1:1 mtROS induction by hyphae was quicker than mtROS induction by yeasts; moreover, hyphae-induced mtROS levels were significantly higher than those induced by yeasts (Fig 1C—left panel and Fig 4B –left panel). The difference in the mtROS activation times could not be observed upon epithelial cells infection with the higher fungal inoculum (MOI 1:5). Indeed, under this experimental condition, the mtROS induction in response to both yeasts and hyphae was overlapping, at least up to 10 h post-infection (Fig 1C—right panel). However, at 12.5 h post-infection hyphae-induced mtROS levels were higher (albeit not significantly) than yeasts-induced mtROS levels (Fig 1C—right panel and Fig 4B—right panel).

### Mitochondrial activation by Ca PCA-2

**Fig 2:**
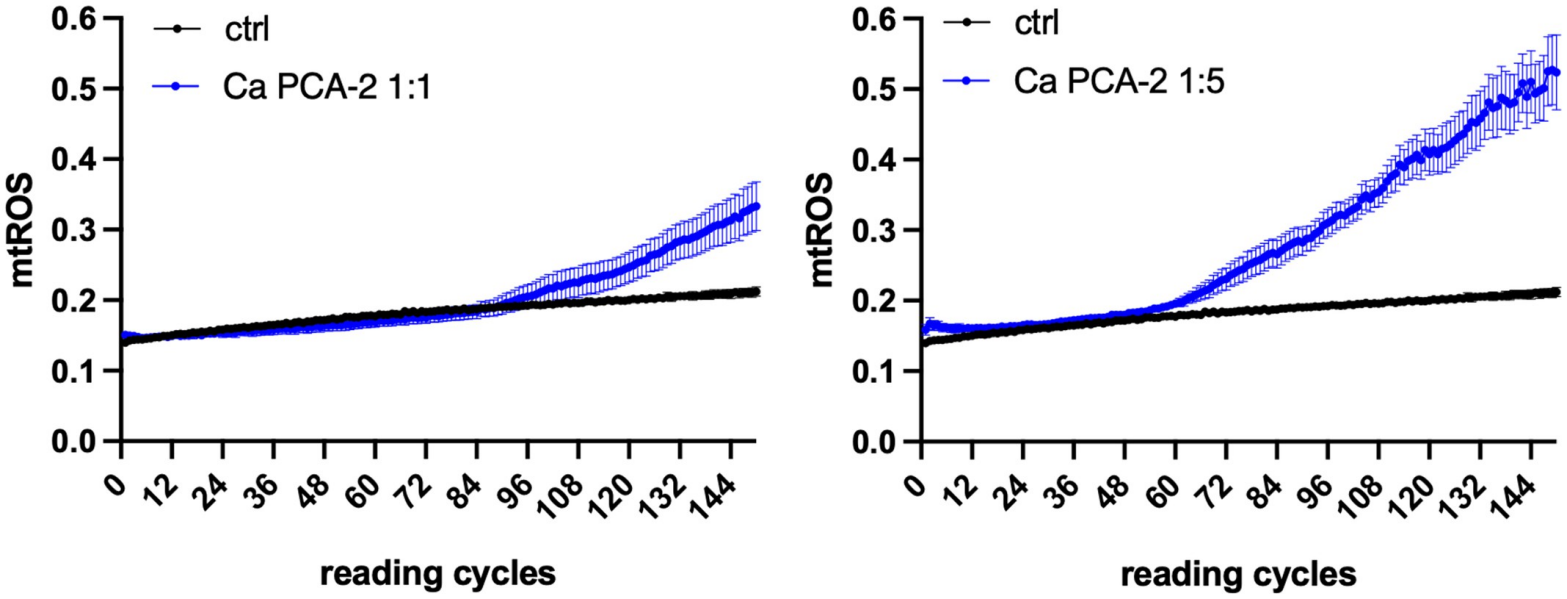
Kinetics of mtROS production by Ca PCA-2 infected RVE. RVE has been infected with Ca PCA-2 either at MOI 1:1 (left panel) or MOI 1:5 (right panel). The mtROS production was evaluated by fluorescence emission after addition of MitoSOX^™^ Red probe, as detailed in Materials and Methods section. Reading cycles were perfomed at 5 minutes intervals. Ctrl = unifected RVE. Data are expressed as mean ± SEM. Data are from at least 3 different experiments performed in triplicate.

To better understand this observation, we challenged RVE with Ca PCA-2 by the same experimental set-up. The Ca PCA-2 strain is unable to switch from yeast to mycelial form [34], so that we could establish only the role played by the fungal load in inducing mtROS. The results shown in Figs 2 and 4A demonstrate that Ca PCA-2 strain could induce mtROS after 7.5 h of infection at MOI 1:1 (92 reading cycles) and after 4.5 h post-infection at MOI 1:5 (54 reading cycles). Therefore, at the same fungal loads, Ca PCA-2 induced mtROS 1 h (MOI 1:5) and 0.5 h (MOI 1:1) later then BLI-Ca yeasts. In addition, the levels of mtROS 12.5 h post-infection were higher at MOI 1:5 than at MOI 1:1, thus demonstrating that higher fungal loads were decisive in increasing levels of mtROS by RVE (Fig 4B).

### Mitochondrial activation by 529L Ca

**Fig 3:**
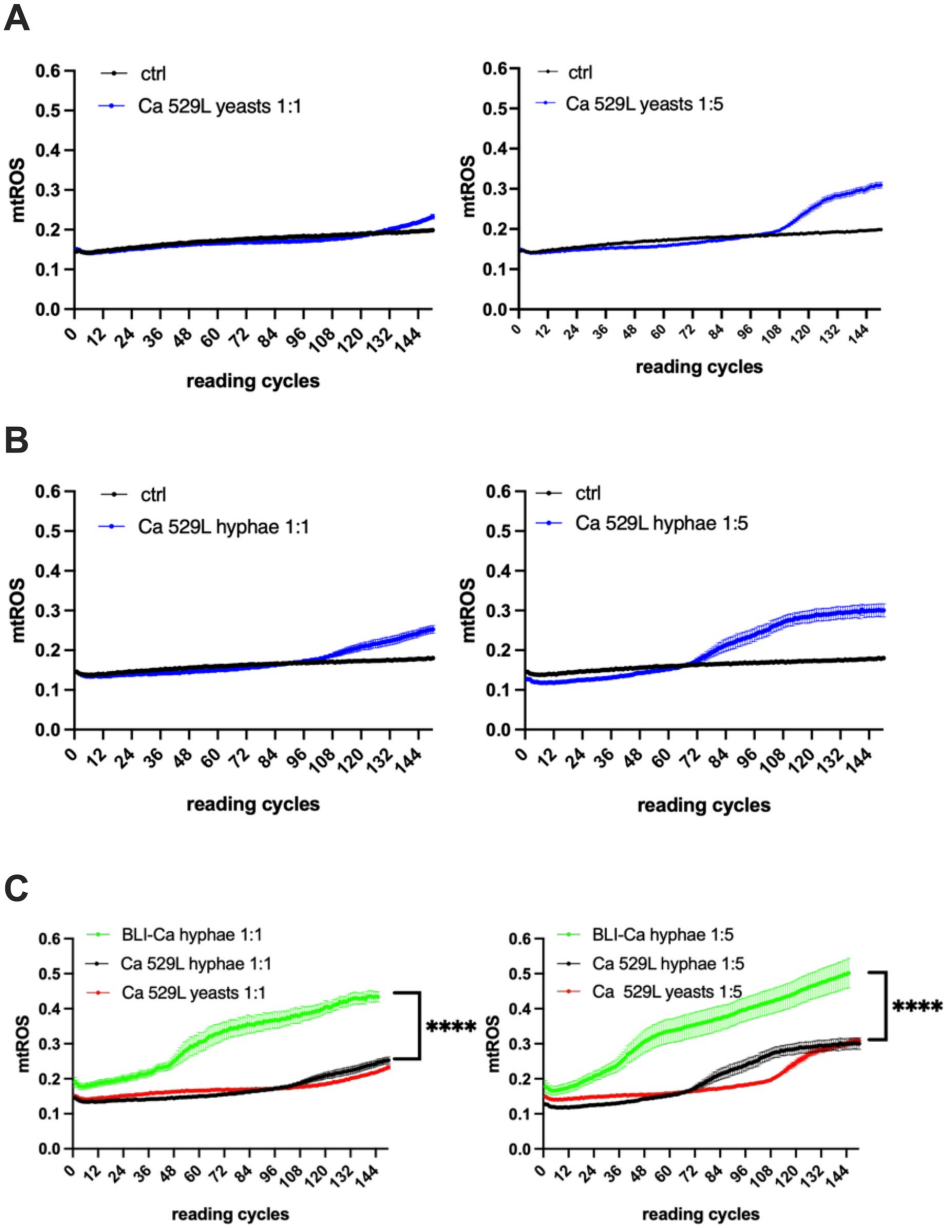
Kinetics of mtROS production by Ca 529L infected RVE. RVE has been infected with Ca 529L in yeasts (**A**) or hyphae (**B**) either at MOI 1:1 (left panels) or MOI 1:5 (right panels). In (**C**) the comparisons between mtROS production by Ca 529L yeasts and hyphae and BLI-Ca hyphae at MOI 1:1 (left panel) and MOI 1:5 (right panel) are shown. The mtROS production was evaluated by fluorescence emission after addition of MitoSOX^™^ Red probe, as detailed in materials and methods section. Reading cycles were perfomed at 5 minutes intervals. Ctrl = unifected RVE. Data are expressed as mean ± SEM. Data are from at least 3 different experiments performed in triplicate. Statistical analysis was performed by using Mann-Whitney U test ****p < 0.0001.

**Fig 4:**
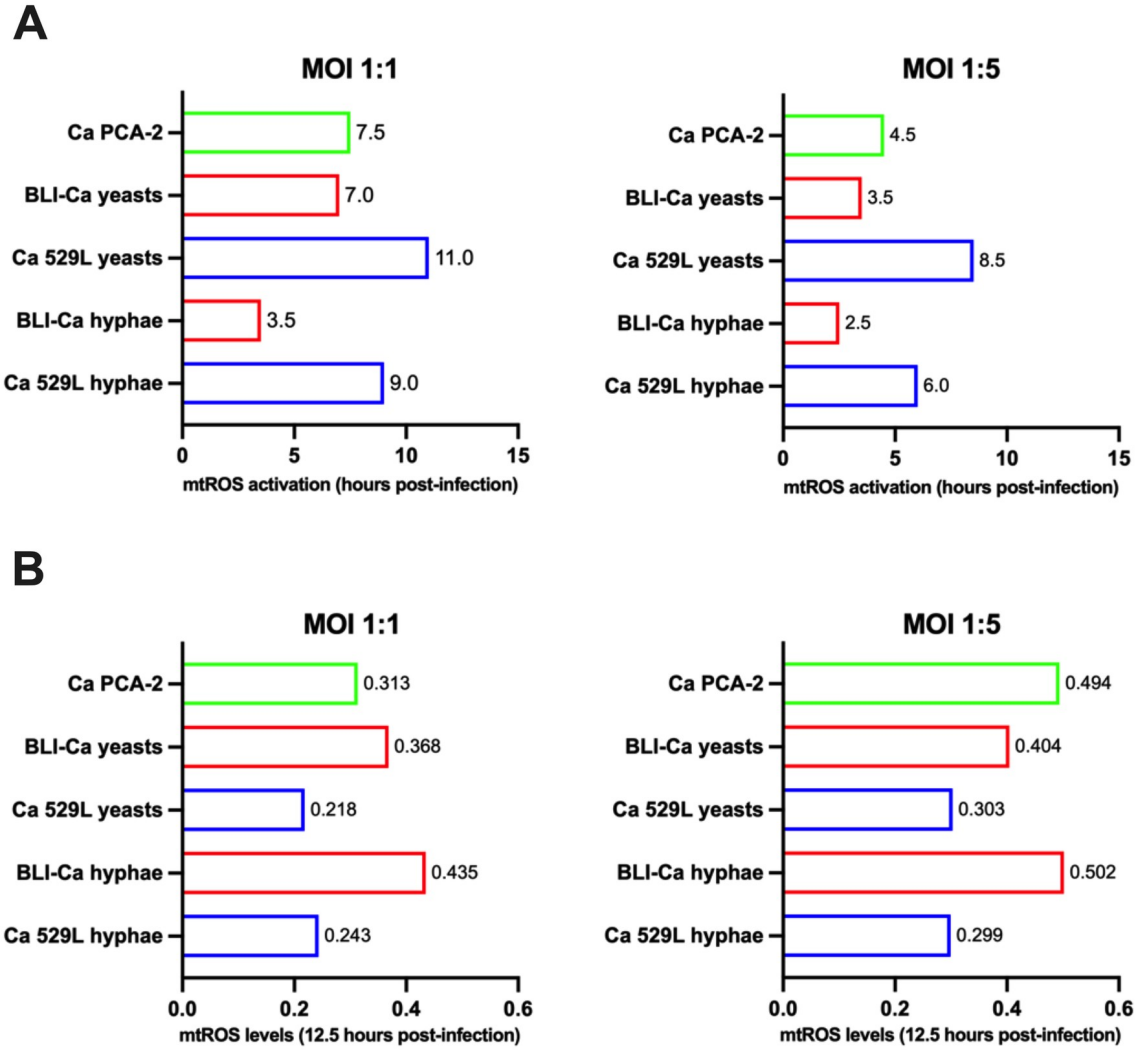
Schematic representation of mtROS activation times and levels. (**A**) Times of induction of mtROS after RVE infection with the different Ca strains at the different experimental conditons are shown at MOI 1:1 (left panel) and MOI 1:5 (right panel). (**B**) The levels of mtROS production reached after 12.5 h of RVE infection with the different Ca strains at the different experimental conditons are shown at MOI 1:1 (left panel) and MOI 1:5 (right panel).

In order to better clarify the role of hyphae in the mtROS induction, we employed the Ca strain 529L, characterized by an impaired production of CL [35]. Our results show that mtROS activation from Ca 529L yeasts at MOI 1:1 could not be observed at least up to 11 h post-infection (132 reading cycles). Differently, mtROS activation from yeasts started after 8.5 h at MOI 1:5 (102 reading cycles) (Figs 3A and 4A). Moreover, we observed mtROS production from hyphae after 9 h at MOI 1:1 (108 reading cycles) and after 6 h at MOI 1:5 (72 reading cycles) (Figs 3B and 4A).

A comparison between BLI-Ca and Ca 529L strains, shows that the former in hyphal form was able to induce significantly higher mtROS levels and more quickly than the latter, both at MOI 1:1 and at MOI 1:5 (Figs 3C and 4B).

The scheme in Fig 4A summarizes the time required by Ca-infected RVE to trigger mtROS production, that was always quicker in BLI-Ca when compared to Ca 529L. Similarly, the scheme in Fig 4B summarizes the levels of mtROS induced at the end of the experiments, i.e., 12.5 h after RVE infection, showing that they were always higher in BLI-Ca than in Ca 529L.

### Cell damage after Ca infection

The assessment of epithelial cell damage is an important datum to define Ca virulence; therefore, we analyzed cell damage after 24 h of RVE infection with yeasts of Ca PCA-2 and yeasts and hyphae of BLI-Ca and Ca 529L both at MOI 1:1 and MOI 1:5. Interestingly, at low fungal burden (MOI 1:1) and in both yeast and hyphal form, Ca 529L induced significantly lower cell damage when compared to BLI-Ca. Notably, at MOI 1:1, Ca 529L yeasts did not induce any damage to RVE (below 5% of cell damage), whereas Ca 529L hyphae induced damage was around 20%, i.e. much lower than BLI-Ca yeasts- and hyphae-induced damage (more than 80%).

**Fig 5:**
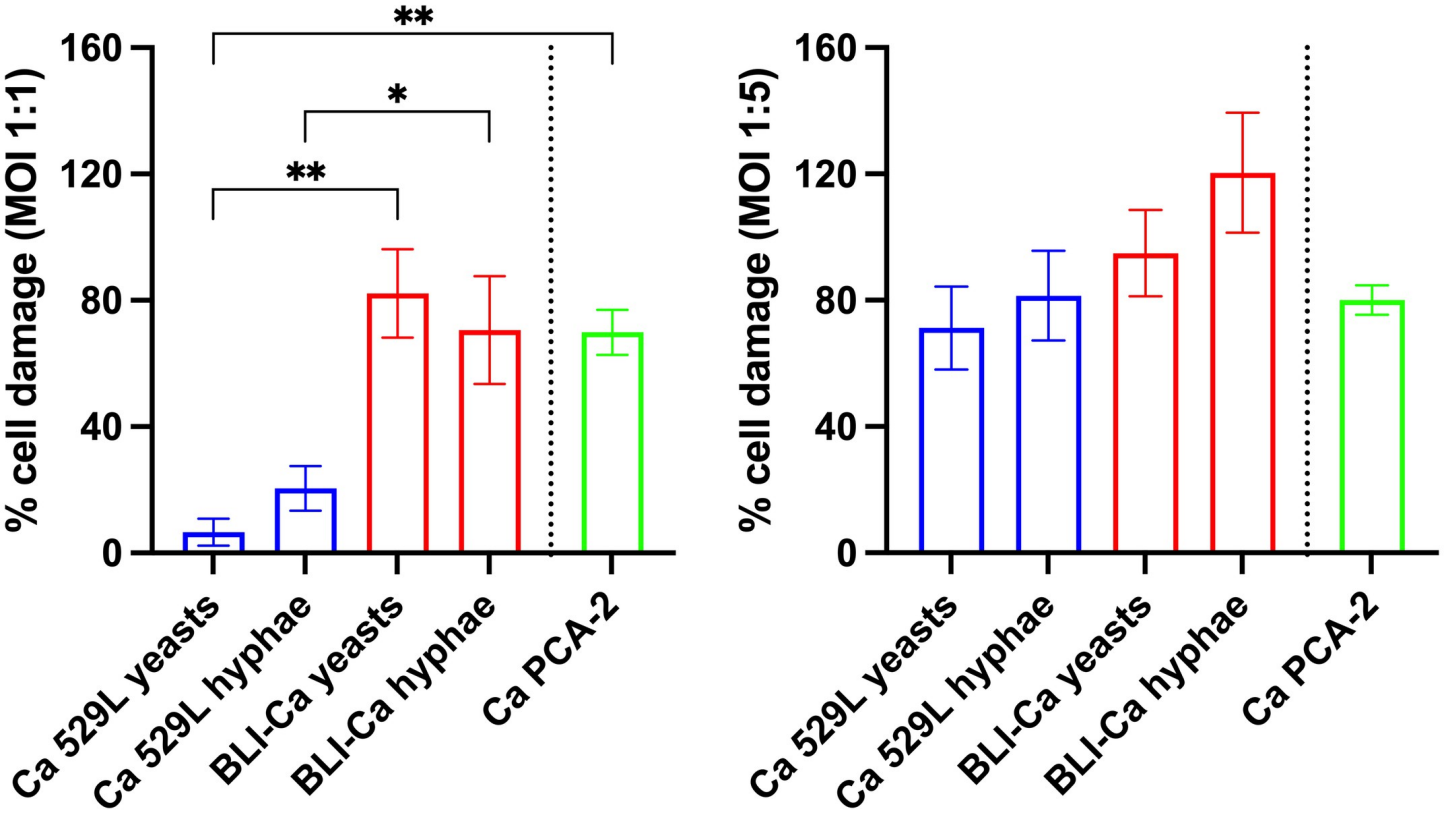
Cell damage after RVE infection with BLI-Ca, Ca 529L and Ca PCA-2. The percent (%) of cell damage after 24h of RVE infection with BLI-Ca and Ca 529L, both in yeasts and hyphal forms, and Ca PCA-2 yeasts at MOI 1:1 (left panel) and MOI 1:5 (right panel) is shown. Statistical analysis was performed by One-Way ANOVA followed by Tukey’s multiple comparisons test. Data are expressed as mean ± SEM. Data are from at least 3 different experiments performed in triplicate. *p < 0.05; **p < 0.01.

Differently, at high fungal load (MOI 1:5) both BLI-Ca and Ca 529L severely damaged the RVE, irrespective of the morphological stage or CL. Ca PCA-2 induced a cell damage comparable to the cell damage induced by BLI-Ca yeasts at both MOIs (Fig 5).

### Role of Ca-induced mtROS in cell damage and fungal growth

**Fig 6:**
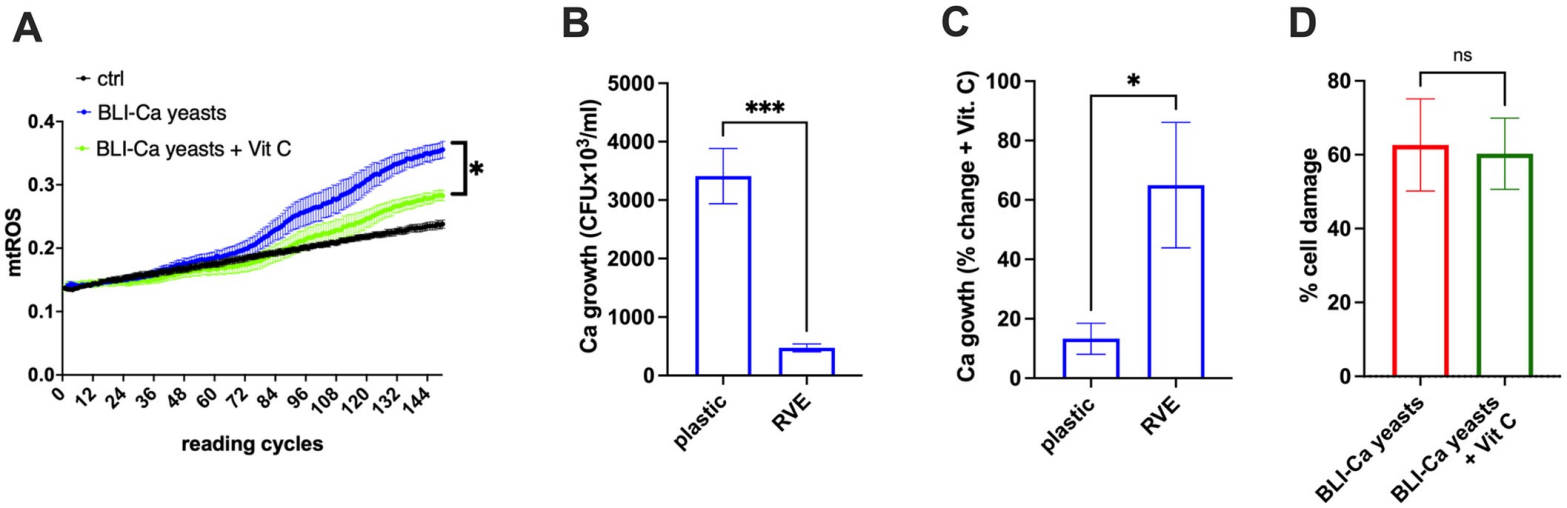
mtROS production, fungal growth and cell damage after RVE infection with BLI-Ca yeasts in the presence of a ROS scavenger. RVE has been infected with BLI-Ca yeasts at MOI 1:1 in the presence or absence of ascorbic acid (Vit C, 1000 μM) used as ROS scavenger. (**A**) The mtROS production was evaluated by fluorescence emission after addition of MitoSOX^™^ Red probe, as detailed in materials and methods section. Recording cycles were perfomed at 5 minutes intervals. Ctrl = unifected RVE. Statistical analysis was performed by using unpaired *t*-test. *p < 0.05. (B) Comparison of BLI-Ca yeasts growth on abiotic surface (plastic) and on RVE. Data are expressed as CFU x 10^3^/ml. (C) Percentage increase of BLI-Ca yeasts growth on abiotic surface (plastic) and on RVE after addition of Vit C in the culture medium. (D) Percentage of cell damage induced by BLI-Ca yeasts on RVE in the presence or absence of Vit C. Data are expressed as mean ± SEM. Data are from at least 3 different experiments performed in triplicate.

Finally, we performed experiments where RVE was infected for 24h with BLI-Ca yeasts at MOI 1:1 in the presence or absence of ascorbic acid (Vitamin C). It is acknowledged that ascorbic acid functions as a potent antioxidant in mitochondria of human cells, therefore in this context, it is used as mtROS scavenger [36]. Our results show that the addition of Vitamin C resulted in a significant reduction of BLI-Ca yeasts induced mtROS (Fig 6A). In the same experimental setting, we quantified fungal growth after RVE infection or culture on an abiotic surface. Our results show that fungal growth was significantly higher on the abiotic surface than on the RVE (Fig 6B).

Notably, by adding Vitamin C to culture medium, a significant increase in fungal growth could be observed in RVE as compared to the fungal growth on abiotic surface (Fig 6C). Moreover, no changes in cell damage after 24h of RVE infection, could be observed after the addition of Vitamin C to the culture medium (Fig 6D).

## Discussion

In healthy women, *C*. *albicans* can behave as a commensal of the vaginal microbiota. Indeed, although Candida cell wall antigens interact with the Pattern Recognition Receptors (PRRs), the epithelial cells tolerate Candida, as well as any other resident microorganism, without triggering any inflammatory response. In this state of tolerance, it is thought that Candida colonizes the mucosal membranes mainly in the form of yeast and with a low fungal load. Nevertheless, if the tolerance threshold is exceeded, this balanced situation breaks down, Candida proliferates (therefore increasing its fungal load) and undergoes mycelial transition [2, 21].

The increased fungal burden, the production of hyphae, as well as toxic molecules such as CL and SAP, have all been described as factors involved in triggering the inflammatory response and the disease onset [2].

The production of mtROS has been recognized as a key mechanism used by the host cells to react against non-self antigens, thus polarizing the immune response [15]. Indeed, mtROS antimicrobial activity after innating immune cells activation has been widely described. Less known is the role exerted by mtROS produced by infected epithelial cells, after fungal infection. Here, we assessed the role of fungal burden, mycelial transition, and CL production in mtROS induction by epithelial cells, and also the potential implication of mtROS production in cell damage and fungal growth.

We started by investigating the role played by the mycelial transition and the fungal burden in the induction of mtROS. Our data show that by employing BLI-Ca, at MOI 1:1, hyphae stimulate mtROS production after 3.5 hours post-infection, whereas at the same MOI, yeasts necessitate of at least 7 hours to induce mtROS and such delay has been shown to be significant (Fig 1C—left panel). Differently, when RVE is infected by a high fungal load (MOI 1:5), also yeasts induce mtROS almost as early as hyphae, suggesting thus that, under such conditions, it is not necessary for Ca to undergo dimorphic transition to induce a quick epithelial activation, as demonstrated by the rapid induction of mtROS. In addition, the short delay in inducing mtROS by yeasts at high fungal load (only 1 h) lacks statistical significance (as shown in Fig 1C—righ panel). To support these data, we employed PCA-2, a Ca strain that is constitutively unable to undergo dimorphic transition. As shown in Fig 2 and in Fig 4A, the mtROS production by such strain closely mirrors the mtROS production by the BLI-Ca yeasts, supporting the idea that fungal load is a key element, other than dimorphic transition, that triggers mitochondrial activation, as demonstrated by the similar levels of mtROS induced at MOI 1:5 by Ca PCA-2 and BLI-Ca hyphae and yeasts (Fig 4B). Even though Ca PCA-2 is not able to form hyphae (and therefore it should be unable to secrete CL) [28], it always stimulates higher levels of mtROS production compared to Ca 529L. This suggestes the Ca PCA-2 might replicate quicker that Ca 529L therefore reaching a higher burden in a shorten time.

Regarding the role of hyphae and pseudohyphae, *in vitro* and *ex vivo* data from the literature describe Ca morphological transition (*i*.*e*., the formation of true hyphae) as the main event responsible for the epithelial activation which drives the immunopathology [37, 38]. Differently, another study supports the idea that pseudohyphae, rather than hyphae, may play a key role in the activation of the inflammatory process that leads to VVC onset [39]. Fungal morphogenesis is an important virulence factor that facilitates invasion of host tissues, escape from phagocytes, and dissemination in the blood stream. The innate immune system is the first line of defense against *C*. *albicans* infections and is influenced by recognition of wall components that vary in composition in different morphological forms [40].

Our data suggest that a high fungal load may be sufficient to trigger mtROS production irrespective of the morphology of the fungus. Such mitochondrial activation may also play a key role in induction of inflammation.

The data presented here show that true hyphae always stimulate more quickly mtROS production by epithelial cells at all the experimental conditions assessed, *i*.*e*., low, and high fungal load.

Ca 529L, characterized by the impaired production of CL [41], has been employed to determine if such toxin plays a role in mithochondrial activation, in addition to morphological transition and fungal burden. Our results have shown that by infecting epithelial cells with Ca 529L, the induction of mtROS occurs much later (and at lower levels) with respect to infections by BLI-Ca. By employing Ca 529L yeasts at MOI 1:1, mtROS induction has not been observed until 11 h post-infection, whereas at MOI 1:5, Ca 529L yeasts have been able to induce mtROS only 8.5 h post-infection. In addition, the mtROS levels induced 12.5 h post-infection by Ca 529L are always lower than those induced by BLI-Ca, irrespective of the MOI. Therefore, by comparing these results with those obtained by infecting the RVE with BLI-Ca, we hypothesize that the yeasts of this latter strain start to undergo morphological transition and the CL produced by the hyphae can accelerate the induction of mtROS. The yeasts of the Ca 529L undergo morphological transition as well, but their impaired production of CL makes them unable to induce mtROS (at low fungal burden) or delays such induction (at high fungal burden). A similar delay in mtROS induction can be observed also with respect to Ca PCA-2. The data obtained by infecting epithelial cells with Ca 529L hyphae once again show a delay in mtROS induction, as compared to the infection with BLI-Ca hyphae (9 h vs 3.5 h at MOI 1:1 and 6 h *vs* 2.5 h at MOI 1:5). Similarly to what observed with Ca 529L yeasts, Ca 529L hyphae induce also lower levels of mtROS when compared to BLI-Ca hyphae. Again, these data point to the relevance of the CL in inducing mtROS. Therefore, according to these results, we hypothesize that CL is a key element to accelerate and potentiate the Ca-induced mitochondrial activity in vaginal epithelial cells.

Interestingly, oral epithelial cells treated with CL showed rapid production of mtROS [18].

Moreover, we have assessed the damage of epithelial cells infected by the BLI-Ca and Ca 529L. In line with studies from other reaserch groups [26], our results show that CL is a key player in Ca-induced epithelial damage, as demonstrated by the low damage levels detected in Ca 529L-infected cells. Interestingly, such effect has been observed only at low fungal burden (MOI 1:1), whereas, at higher fungal burden (MOI 1:5), the epithelial cell damage reaches higher levels, irrespective of the CL production and fungal morphology. Therefore, CL is necessary to induce damage at low fungal burden indicating that, in such condition, the simple presence of hyphae is not enough to damage epithelial cells.

Mitochondrial activity after *C*. *albicans* infection was also tested in the presence of the mtROS scavenger ascorbic acid (Vitamin C). Interestingly, we show that the mtROS scavenging leads to an increased fungal growth only when *C*. *albicans* is infecting RVE but not when the fungus is growing on an abiotic surface. This result suggests that mtROS may be a key element of vaginal epithelial cell response to *C*. *albicans*.

Collectively, our data show that mtROS are differentially regulated by vaginal epithelial cells according to fungal burden, presence or hyphae and secretion of CL. It has been demonstrated that oral epithelial cells respond to CL treatment with a rapid production of mtROS, disruption of mitochondria activity and mitochondrial membrane potential, ATP depletion and cytochrome c release, demonstrating that oral epithelial cells respond to CL by triggering numerous cellular stress responses [18]. To the best of our knowledge, this is the first work that shows how a Candida strain whose production of candidalysin is impaired (Ca 529L), is weakened in its capacity to induce mtROS by RVE.

In line with to what observed in oral epithelial cells after CL treatment, our future work will be devoted to unravelling the precise role of mtROS activation in the interplay between vaginal epithelial cells and Candida.

## Materials and methods

### Microbial strains and growth conditions

The bioluminescent strain of *Candida albicans* CA1398 carrying the bioluminescence ACT1p-gLUC59 fusion product (BLI-Ca) [42] was chosen in order to use the relative luminescence units (RLU) to count the preformed hyphae, as detailed below in “Establishment of a standard curve to count BLI-Ca hyphae”. The *Candida albicans* strain PCA-2 (Ca PCA-2), was employed because it is unable to undergo dymorphic transition [34]. The *C*. *albicans* strain 529L (Ca 529L) was employed because of its impaired production of candidalysin [35]. The strains have been stored in frozen stocks at -80°C in Sabouraud Dextrose Broth (Condalab, Spain) supplemented with 15% glycerol. Every six months strains were reactivated, subcultured and new frozen stocks were prepared. After thawing, the fungi were grown in liquid YPD medium (Yeast extract—Peptone—Dextrose, Scharlab S.L., Spain) and incubated at 37°C under aerobic conditions for 24 h. Fungal cultures were maintained by passages onto SAB agar biweekly. For infection we employed two different protocols to produce yeasts or hyphae as detailed below.

### Production of *C*. *albicans* yeasts

For the infection with yeasts, a loop of BLI-Ca, Ca PCA-2, or Ca 529L were seeded in 5 ml of YPD broth and incubated at 30°C under agitation overnight. Then fungi were washed with PBS, counted with an heamocitometer by excluding dead cells with Trypan blue staining and resuspended to a working strength MOI 1:1 or 1:5 in DMEM-5% heat inactivated FBS with respect to vaginal cells.

### Production of *C*. *albicans* hyphae

For the infection with hyphae, hyphal fragments were produced according to a protocol established by Hopke and Wheeler [43]. According to this protocol, chosen because it had been specifically set up for *C*. *albicans*, BLI-Ca and Ca 529L were seeded in 5 ml of YPD broth and grown at 30°C under agitation overnight. Then fungi were washed, counted by heamocitometer to exclude dead cells with Trypan blue staining and resuspended in 35 ml of RPMI-1640 at concentration of 2.5 x10^6^/ml and distributed in 6 tubes, each containing 5 ml of fungal suspensions. Such tubes were incubated overnight under agitation at 30°C. After incubation, fungi were washed, all the pellets were put together and resuspended in 1 ml of PBS.

To count BLI-Ca hyphae, 1 μl of coelenterazine (Synchem, 1 mg/ml) was added to 100 μl of BLI-Ca suspension and measured by Fluoroskan (ThermoFischer Scientific). The use of coelenterazine was necessary because such molecule is the natural substrate of luciferase enzyme occurring on the cell wall of BLI-Ca [42]. Only values of Relative Luminescence Units (RLU) substracted of the blank (a well containing 100 μl of PBS and 1 μl of coelenterazine), were used to calculate the CFU in reference to a standard curve previously established (as detailed below).

The OD_570_ values corresponding to 100 μl of either BLI-Ca or Ca 529L hyphae were analyzed by a spectrophotometer (SunRise Tecan). The CFU of Ca 529L were calculated by comparison to the BLI-Ca CFU values that had been estimated from the standard curve.

### Establishment of a standard curve to count BLI-Ca hyphae

To quantify hyphal fragments by using luminescence emission, a calibration curve was generated. Serial dilutions of BLI-Ca hyphae were prepared. One hundred μl of each dilution was seeded in Sabouraud agar plates, incubated for 24–48 h at 30°C and then the CFU were counted. The same dilutions were dispensed in the wells of a 96-well black microtiter plate (100 μl/well) and luminescent signal was read at Fluoroskan after addition of coelenterazine (1 μl/well, 1 mg/ml). The generated calibration curve allowed an estimation of the CFU from the RLU.

### A-431 epithelial cells

The human epithelial A-431 cell line derived from a vaginal epithelial squamous cell carcinoma was used. The cell line was puchased from LGC Standards, catalogue number ATCC-CRL-1555. This cell line is widely employed to produce monolayers or multilayers mimicking the vaginal epithelium [4, 32]. These cells were cultured in DMEM medium (Dulbecco’s Modified Eagle Medium, PAN Biotech) supplemented with L-glutamine (2 nM) (Euroclone SpA, Italy), penicillin (100 U/ml) (Euroclone SpA, Italy), streptomycin (100 μl/ml) (Euroclone SpA, Italy), ciprofloxacin (20 mg/ml) (Euroclone SpA, Italy) and heat-inactivated Fetal Bovine Serum (h.i. FBS, 10% or 5%, SIGMA-Aldrich, USA); specifically, medium containing 10% h.i. FBS was used to allow the establishment of the Reconstituted Vaginal Epithelium (RVE), whereas medium containing 5% h.i. FBS was used for the infection. The cell line was kept in culture by-passages in fresh medium twice a week and incubated at 37°C and 5% CO_2_.

### Reconstituted vaginal epithelium (RVE) infection and mtROS production analysis

A-431 cells 5x10^5^/ml (1x10^5^ in 200 μl/well) were grown in DMEM + 10% h.i. FBS for 5 days in a 96-well black-trasparent plates. At days 2 and 4 the medium was replaced with fresh medium. RVE was then infected with the different Ca strains (yeasts or hyphae) MOI 1:1 or 1:5. For the determination of mtROS production, MitoSOX^™^ Red (2.5 μM/well) (Invitrogen^™^, ThermoFisher Scientific) was added in each well immediately after infection. Then, the plates were kinetically measured every 5 min (5 min = 1 reading cycle) by Fluoroskan under stable temperature of 37°C. The fluorescence emission was analyzed at excitation/emission 544/590.

In selected experiments, the antioxidant molecule ascorbic acid (Vitamin C, 1000 μM; Sigma Aldrich) [44] was added during RVE infection with BLI-Ca yeasts (MOI 1:1) and then mtROS production has been kinetically analyzed as above described.

### Analysis of cell damage

RVE was infected with Ca PCA-2 yeasts, BLI-Ca and Ca 529L yeasts or hyphae at both MOI 1:1 and MOI 1:5. Twenty-four hours post-infection, cell damage was quantified by the analysis of Lactate dehydrogenase (LDH) release in the growth medium by using a specific colorimetric kit (Abcam).

In selected experiments, RVE was infected with BLI-Ca yeasts (MOI 1:1) in the presence or absence of ascorbic acid (Vitamin C, 1000 μM; Sigma Aldrich). After 24h of infection, cell damage was assessed by LDH kit.

### Fungal growth

RVE was infected with BLI-Ca yeasts (MOI 1:1) in the presence or absence of ascorbic acid (Vitamin C, 1000 μM; Sigma Aldrich). After 24h of infection, cells were lysed with 0.1% Triton X-100 and serial dilutions were performed for CFU counting. As a control, the same concentration of BLI-Ca yeasts was grown under the same experimental conditions but without RVE.

### Statistical analysis

Shapiro-Wilk test was used to analyze the distribution of data within experimental groups. All statistical analyses were performed by using GraphPad Prism 10.3 software.

The statistical analysis of kinetic data was performed following the “GraphPad guide to comparing dose-response or kinetic curves” [45]. For each kinetic curve obtained in the experimental procedures, the Area Under the Curve (AUC) was calculated to summarize the curve into a single value. Subsequently, statistical analysis was performed on the AUC values of each experimental group using unpaired *t*-test or a Mann-Whitney test (see Figure legends), depending on the distribution of data. Statistical differences between groups for non-kinetic data were assessed by One-Way ANOVA followed by Tukey’s multiple-comparisons test.

Values of *p<0.05, **p<0.01, ***p<0.001 and ****p<0.0001 were considered statistically significant.
